# Limited significance of repeated long-term radiological and hormonal examination in nonfunctioning adrenal incidentalomas

**DOI:** 10.1590/S1677-5538.IBJU.2018.0235

**Published:** 2019-07-27

**Authors:** Masayuki Tasaki, Takashi Kasahara, Itsuhiro Takizawa, Kazuhide Saito, Tsutomu Nishiyama, Yoshihiko Tomita

**Affiliations:** 1 Division of Urology, Department of Regenerative and Transplant Medicine, Graduate School of Medical and Dental Sciences, Niigata University, Niigata, Japan

**Keywords:** Adrenal incidentaloma [Supplementary Concept], Adrenalectomy, Radiology

## Abstract

**Purpose:**

The purposes of the present study were to evaluate growth rate of nonfunctioning adrenal incidentalomas (AIs) and their development to hormonal hypersecretion on follow-up.

**Materials and methods:**

A retrospective study was conducted from the electronic medical records. A total of 314 patients were diagnosed with adrenal tumors between 2000 and 2016. After excluding patients who had overt adrenal endocrine disorders or whose adrenal tumors were clinically diagnosed as metastatic malignancies, we investigated 108 patients with nonfunctioning AIs including characteristics, the treatment, the way of follow-up and pathology.

**Results:**

Fifteen patients received immediate adrenalectomy because of the initial tumor size or patient's preference. Pathological examination revealed malignancy in 2 patients. In the remaining 93 patients, radiological examinations were performed periodically. Tumor enlargement of ≥ 1.0cm was observed in 8.6% of the patients who were followed up as nonfunctioning AIs with a median follow-up period of 61.5 months (range: 4-192). Eleven patients underwent adrenalectomy. On the pathological examinations, all of the tumors, which showed a size increase, were diagnosed as benign tumors. Regarding the followed up patients without adrenalectomy, only 2.4% of the patients had tumor enlargement during the prolonged follow-up. Furthermore, none of the patients developed hormonal hypersecretion or clinical signs such as obesity, glucose intolerance or poorly controlled hypertension.

**Conclusions:**

Tumor enlargement of AIs did not correlate with malignancy. The value of repeat radiological and hormonal examinations may be limited in the long-term follow-up of patients whose AIs are not enlarged.

## INTRODUCTION

Adrenal incidentaloma (AI) is defined as an asymptomatic adrenal mass detected on imaging not performed for suspected adrenal disease. The prevalence of AIs has been reported to be as high as 8% in an autopsy series and 4% in a radiologic series (1, 2). With advances in imaging technology and the widespread application of abdominal imaging procedures, the rate of diagnosing AIs is expected to continue increasing. Given that the occurrence of AIs increases with age (3), the management of AIs will also become a growing clinical interest, especially in a rapidly aging society such as Japan. However, most of AIs are nonfunctioning cortical adenomas that are benign and do not cause classical clinical signs and symptoms of hormone excess syndromes (4, 5). The optimal frequency and duration of follow-up for patients with nonfunctioning AIs are uncertain. Long-term surveillance, including radiological and hormonal examinations, may be associated with significant emotional distress (6) and financial costs (7) in these patients, even though the majority of them are found to be healthy. In this study, we evaluated growth rate of nonfunctioning AIs and the development of hormonal hypersecretion on follow-up to explore the management of AIs including surgery and the value of repeated long-term examination.

## MATERIALS AND METHODS

### Patients

**Figure 1:**
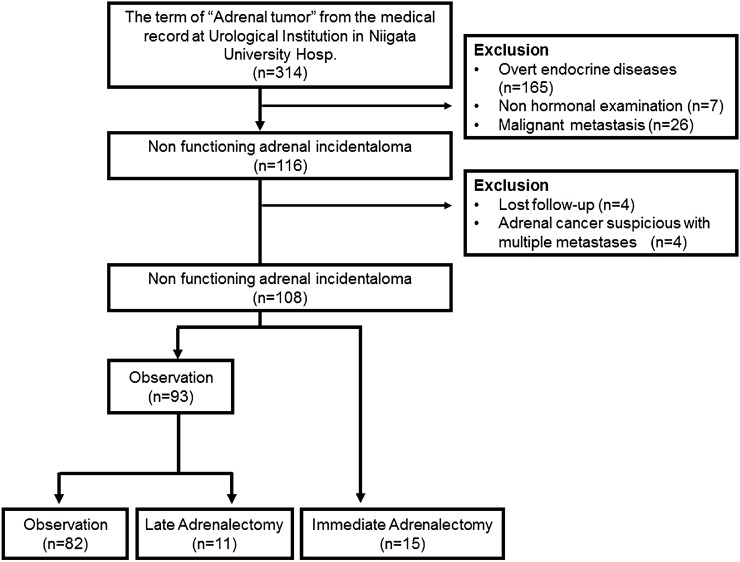
Flow chart of patients.

We used the search term “adrenal tumor” from the electronic database between 2000 and 2016 at the Department of Urology of our institute. A total of 314 patients were found from the database whose adrenal tumors were pointed out incidentally or by the examination for overt endocrine diseases or as adrenal metastases from other primary malignancies. Overt endocrine diseases were diagnosed in 165 patients including primary hyperaldosteronism (n = 68), pheochromocytoma (n = 54), Cushing's syndrome (n = 30), and subclinical Cushing's syndrome (n = 13). Hormonal examinations were not performed in 7 patients at diagnosis. Twenty-six patients whose adrenal tumors were discovered during periodic radiological check-up (clinically diagnosed as malignant metastases) were excluded from the study. The remaining 116 patients were diagnosed as having hormonally nonfunctioning AIs. After excluding 4 patients who were lost to follow-up and 4 patients who were clinically diagnosed as adrenal carcinomas with multiple metastases, 108 patients with nonfunctioning AIs were retrospectively analyzed in this study (Figure-1). Fifteen patients underwent immediate adrenalectomy (Adx) (immediate-Adx group) because of the tumor size ≥ 4.0cm at initial investigation or because the patients strongly wished surgical removal. Eleven patients received late Adx during follow-up (late-Adx group) mainly because of tumor enlargement. The other 82 patients were followed without Adx (observation group) but with periodic computed tomography (CT).

### Hormonal assessment

Hormonal evaluation at diagnosis consisted of morning plasma levels of adrenocorticotropic hormone (ACTH) and cortisol, plasma renin activity, plasma concentrations of aldosterone, dehydroepiandrosterone sulphate (DHEA-S) and catecholamines, and / or 24h urine collection of catecholamines or their metabolites. Some patients received dexamethasone suppression tests at diagnosis. After initial evaluation, the patients were followed by multiple urologists at our hospital. The decisions to repeat hormonal examinations and screening intervals during follow-up evaluation were made by each urologist. Basically, plasma renin activity and plasma concentrations of ACTH, cortisol, aldosterone and catecholamines were measured every 4-12 months in all patients except for immediate-Adx group. Urine catecholamines or their metabolites were assessed in selected patients.

Plasma cortisol and plasma renin activity were measured by enzyme immunoassay with normal range 6.4-21.0μg / dL and 0.2-3.9ng / mL / hr, respectively. Plasma ACTH was measured by electrochemiluminescence immunoassay with normal range 7.2-63.3pg / mL, Plasma aldosterone was measured by RIA with normal range 35.7-240pg / mL. DHEA-S was measured by chemiluminescent enzyme immunoassay. Plasma and urine catecholamines and their metabolites were measured by high performance liquid chromatography. Hematological profile, lipid profile, serum electrolytes, glucose, creatinine / eGFR, and liver function tests were determined by routine laboratory methods.

### Radiological investigation

All patients received CT scans at diagnosis and six months later to check mass enlargement. Then, periodic CT follow-up evaluations were performed every 6-12 months. Lesions were classified as benign if they were small (< 4.0cm) and had radiological features consistent with adenoma / hyperplasia (homogeneous, well circumscribed, lipid rich with radiodensity of < 10 Hounsfield units (HU) on unenhanced CT) or if they had other typical benign characteristics such as a thin-walled cyst, myelolipoma, and adrenal hemorrhage. Magnetic resonance imaging (MRI) and / or enhanced CT were used for patients with lipid-poor (HU ≥ 10 on unenhanced CT), heterogeneous, or large (≥ 4.0cm) tumors. MRI and enhanced CT were used in 15 and 48 patients, respectively. Contrast-enhanced CT examinations were performed at 1 minute (early contrast-enhanced CT) and 15 minutes (delayed contrast-enhanced CT) after administration of contrast material. An absolute contrast enhancement washout was calculated by the following equation: (= 100 x (HU early - HU delayed) / (HU early - HU unenhanced)). An absolute contrast enhancement washout > 60% was assumed to suggest that an adrenal lesion was benign (8). When re-imaging of CT or MRI didn't show enlargement of tumors, they were diagnosed as benign.

### Statistical analysis

Variables were analyzed for normal distribution using the Kolmogorov-Smirnov test. Continuous variables are expressed as medians with range and were compared by using the Mann-Whitney U test. Categorical variables were compared using the Chi-squared test. P < 0.05 was regarded as the difference between significant groups.

## RESULTS

The main clinical characteristics of the 108 patients with nonfunctioning AIs consisting of 3 groups are summarized in Table-1. The mean age at diagnosis and sex were not significantly different among the groups. Left-sided adrenal tumors were more common in all groups. The median follow-up periods were 63.0 and 35.0 months in the observation and late-Adx groups, respectively. Tumor size at diagnosis was significantly larger in the immediate-Adx group than in the other groups (P < 0.001). The ratio of tumors of < 10HU on unenhanced CT was higher in the observation group (61.0%) than in the late- (36.4%) and immediate- (20.0%) Adx groups, but this difference was not significant. However, the percentage of small tumors (< 4.0cm) was significantly higher in the observation group than in the other groups (P < 0.001). Tumor enlargement of ≥ 1.0cm during follow-up was more frequent in the late-Adx group (54.6%) than in the observation group (2.0%). Table-2 shows the summary of immediate-Adx. Two patients (cases 3 and 12) with a tumor size of < 4.0cm underwent immediate-Adx because they had a previous history of malignancy and hoped to remove their adrenal tumors in order to rule out malignancy. In two patients (cases 14 and 15), pathological examination revealed their tumors to be malignant. Table-3 summarizes the late-Adx group. Five patients with small tumors (< 4.0cm) at diagnosis exhibited a remarkable tumor enlargement during follow-up (cases 16, 17, 19, 20 and 21). Radiological follow-up was performed in 4 patients with a tumor size of ≥ 4.0cm because the CT findings indicated those tumors to be benign (cases 22-25). During follow-up, those tumors gradually increased in size, and subsequently late-Adx was conducted. On the pathological examinations, however, all of the tumors, which showed a size increase, were diagnosed as benign tumors. Case 26 is an exceptional case. She was initially examined by CT scan because of her right abdominal pain that showed right adrenal hematoma. By periodic CT scans, the tumor decreased in size with the shrinkage of hematoma, and meanwhile, it showed appearances suggestive of a malignant tumor. Although Adx was tried, it could not be removed due to strong adhesions, and hence, only core biopsy was conducted and showed adrenal carcinoma.

**Table 1:** Characteristics of non-functioning adrenal incidentalomas.

	Observation (n=82)	Late-Adx (n=11)	Immediate-Adx (n=15)	P-value
Age*, mean±SD	62.5 (29.0-89.0)	56.0 (31.0-70.0)	60.0 (30.0-79.0)	0.190 1
Male, n (%)	45 (54.9)	4 (36.4)	7 (46.7)	0.407 1
Tumor laterality (Left / Right / Both)	50/23/9	8/3/0	9/5/1	–
Follow-up period (M), median (range)	63.0 (7-192)	35.0 (4-90)	NA	0.019 2
Tumor size * (cm), median (range)	2.2 (0.5-8.0)	3.3 (0.7-7.4)	4.7 (2.1-19.3)	<0.001 1
CT density < 10HU on unenhanced CT *, n (%)	50 (61.0)	4 (36.4)	3 (20.0)	0.468 1
Tumor size < 4.0cm *, n (%)	72 (87.8)	6 (54.6)	3 (20.0)	<0.001 1
Tumor enlargement 1cm during follow-up, n (%)	2 (2.4)	6 (54.6)	NA	<0.001 1

* Initial evaluation;,

1 Kruskal Wallis;

2 Mann-Whitney's U-test / **Adx =** adrenalectomy; **HU =** Hounsfield unit; **NA =** not applicable.

**Table 2:** Tumor size, co-existence of malignancy, and pathology in immediate adrenalectomy group.

case	Size	co-existing malignancy	Pathology
1	5.0 cm		Cortical adenoma
2	9.0 cm		Schwannoma
3	3.3 cm	Liver cancer tongue cancer	Cortical hyperplasia
4	4.0 cm		Mature teratoma
5	5.0 cm		Cortical adenoma
6	4.0 cm	Gastric cancer Prostate cancer	Hemangioma
7	19.3 cm		Hemangioma
8	4.0 cm		Cortical hyperplasia
9	4.7 cm		Hematoma
10	5.2 cm		Ganglioneuroma
11	4.3 cm		Cortical adenoma
12	2.1 cm	Gastric cancer	Cortical adenoma
13	8.3 cm		Cortical adenoma
14	3.0 cm		Neuroendocrine carcinoma
15	12.0 cm		Adrenal carcinoma

**Table 3:** Tumor enlargement, co-existence of malignancy, and pathology in late adrenalectomy group.

case	Tumor enlargement (Follow-up period)	Co-existing malignancy	Pathology
16	0.7 cm → 3.8 cm (5Y)		Hemangioma
17	2.0 cm → 3.0 cm (7M)	Prostate	Cortical adenoma
18	2.3 cm → 2.4 cm (4M)	Prostate, Lung, Gastric	Cortical adenoma
19	2.6 cm → 4.3 cm (5Y9M)		Cortical adenoma
20	3.0 cm → 5.3 cm (3Y6M)	Kidney	Hematoma
21	3.5 cm → 4.3 cm (6Y4M)		Pseudocyst
22	4.0 cm → 4.5 cm (1Y)		Hyperplasia
23	4.5 cm → 6.1 cm (3Y)		Myelolipoma
24	5.3 cm → 7.0 cm (3Y)		Cortical adenoma
25	5.8 cm → 6.2 cm (5M)		Ganglioneuroma
26	7.4 cm → 5.0 cm (11M)		Adrenal carcinoma

**M** = Months; **Y** = Years

**Figure 2:**
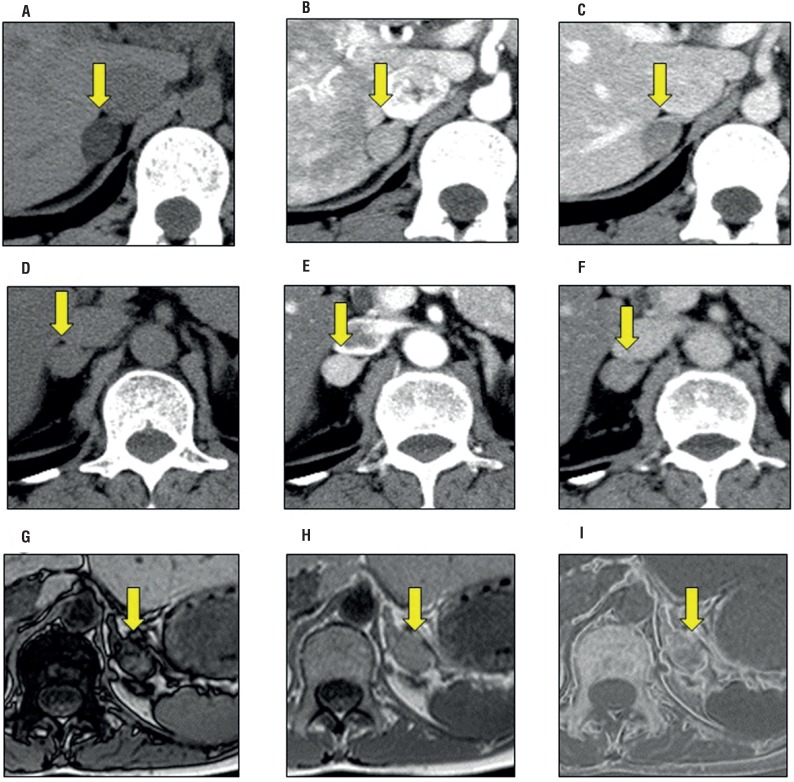
Radiological images of adrenal adenoma. a-c: Lipid rich typical adrenal adenoma. (a) Unenhanced CT, (b) Early phase on contrast enhanced CT, (c) Delayed phase on contrast enhanced CT. d-f: Lipid poor adrenal adenoma. (d) Unenhanced CT, (e) Early phase on contrast enhanced CT, (f) Delayed phase on contrast enhanced CT. g-i: Lipid poor adrenal adenoma. (g) Out-of-phase MR image, (h) in-phase MR image, (i) Chemical shift subtraction MR image. Arrows indicate the adrenal tumors.

We showed the representative radiological images of benign adrenal adenomas in Figure-2. Unenhanced CT showed homogeneous, well circumscribed, lipid rich with radiodensity of 2HU in typical adrenal adenoma (Figure-2a). IV contrast enhanced attenuation of 139HU in early phase (Figure-2b) and 46HU in delayed phase (Figure-2c). An absolute contrast enhancement washout was 67.8%. Lipid poor adrenal adenoma was shown in Figure-2d which had 30HU on unenhanced CT. IV contrast enhanced attenuation of 160HU in early phase (Figure-2e) and 70HU in delayed phase (Figure-2f). An absolute contrast enhancement washout was 69.2%. Chemical shift imaging on MRI was shown in Figure-2g-i. This mass had 35HU on unenhanced CT (data not shown). Signal intensity lost on out-of-phase when compared to in-phase imaging signified the presence of intracellular lipid (Figures 2g and h). Chemical shift subtraction MR image showed marked signal increase throughout mass, indicating lipid content (Figure-2i). All these AIs were not surgically removed, however, the sizes did not change during the follow-up periods, suggesting benign adrenal adenomas.

**Figure 3:**
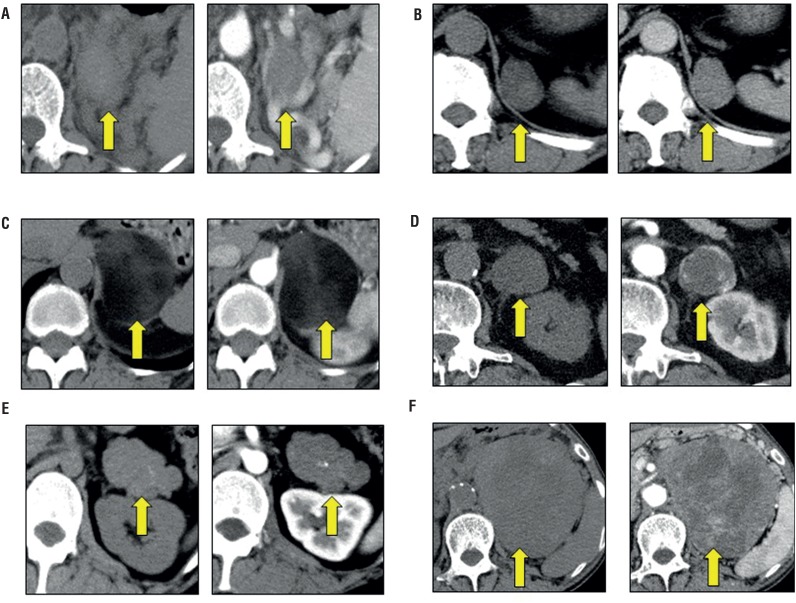
CT images of surgically resected AIs. (a) Adrenal hematoma. (b) Pseudocyst. (c) Myelolipoma. (d) Adrenal hemangioma. (e) Ganglioneuroma. (f) Adrenal carcinoma. Arrows indicate the adrenal tumors.

Figure-3 shows CT findings of adrenal tumors which were surgically resected in this study. Adrenal hematoma was a poor-enhancing round shape tumor (Figure-3a). Pseudocyst was a well-defined hypo-attenuating mass on unenhanced CT and slightly enhanced following injection of contrast medium (Figure-3b). Myelolipoma was a large round soft-tissue attenuating mass containing predominantly fatty components and poor-enhancing on contrast enhanced CT (Figure-3c). Adrenal hemangioma was a well-defined hypo-attenuating mass on unenhanced CT and had heterogeneous enhancement after contrast medium injection (Figure-3d). Ganglioneuroma was a hypo-attenuating relative to muscle on both unenhanced and contrast enhanced CT with the presence of calcification (Figure-3e). Adrenal carcinoma was a large mass with central necrosis and had heterogeneous enhancement following injection of contrast medium (Figure-3f).

None of the patients developed hormonal hypersecretion in periodic blood testing or clinical signs such as obesity, glucose intolerance or poorly controlled hypertension.

## DISCUSSION

The optimal frequency and duration of follow-up for patients with nonfunctioning AIs are uncertain. Follow-up is considered to be necessary for two reasons: (i) to determine if there is a rapid increase in the size of a benign-appearing mass, a finding that would prompt surgery to remove a lesion that may be a cancer, and (ii) to detect the appearance or progression of hormonal activity of the adrenal tumors, a finding that would prompt surgery to avert complications driven by hormonal excess.

According to the NIH Statement (5), the size and appearance of an adrenal mass on CT or MRI may help distinguish between benign and malignant lesions. The available data suggest that nearly all lesions smaller than 4cm are benign. In the present study, 80% of the immediate-Adx group had adrenal tumors ≥ 4.0cm and primary adrenal cancers were determined in two patients, both of whom died of recurrence within a year.

Except for the immediate-Adx group, eight patients (8.6%) exhibited increases in tumor size of > 1.0cm during follow-up, and six patients received Adx; however, pathological examination showed no malignancy. Tumor enlargement did not correlate with malignancy and had questionable value in the present study. In a systemic review, the estimated pooled risk for developing malignancy in AIs was 0.2% (95% confidence interval: 0.0-0.4), which were compatible with a risk of fatal cancer attributed to cumulative radiation exposure with repeated CT scans (9). In two cohort studies, one case of adrenal non-Hodgkin lymphoma and one case of renal cancer metastasis were detected. However, in the first case, initial evaluation was not consistent with benign characteristics (10). In the second case, it was unclear whether the adrenal tumor was detected incidentally or during follow-up for cancer (11). We suggest that initial evaluation is more important than repeated radiological follow-up to rule out adrenal malignancy. The European Society of Endocrinology and the European Network for the Study of Adrenal Tumors guidelines suggest that if unenhanced CT findings are consistent with a benign adrenal tumor (radiodensity ≤ 10HU, homogenous mass, and tumor size < 4.0cm), no further imaging is required (12). However, approximately 20%-30% of adrenal adenomas are lipid poor and have an unenhanced CT attenuation value of > 10 (13). The combination of unenhanced CT and contrast washout values of adrenal tumors can assist in the characterization and differentiation of adenomas from other adrenal tumors with 98% sensitivity and 92% specificity (14–16). In the present study, contrast washout value of adrenal tumors by contrast CT was not examined in all cases. However, if re-imaging of unenhanced CT showed non-enlargement of adrenal tumors, majority of AIs were unharmful for the patients. No one developed malignancy in the patients who followed without Adx (observation group) in our study with 5 years of median follow-up period. If there is the possibility of adrenal malignancy in the initial assessment, radiological evaluation should be repeated after 3-6 months (17). MRI and 18 fluoro-2-deoxyglucose positron emission tomography (FDG-PET) could be options. Loss of signal intensity between in- and out-of-phase images in MRI (18), and the maximum standardized uptake value (SUVmax) and the ratio of SUVmax in the adrenal gland compared with the liver (adrenal-liver ratio) in FDG-PET (19) are useful for differentiating benign from malignant AIs.

In the present study, none of the patients with negative adrenal gland disorders at an initial evaluation developed hormonal hypersecretion during the prolonged follow-up. No one developed clinical signs such as obesity, glucose intolerance or poorly controlled hypertension. Our results are consistent with previous investigations by Terzolo et al. (20). The pooled risk of developing clinically relevant hormonal excesses (e.g., primary aldosteronism, Cushing's syndrome, and pheochromocytoma) is < 0.3% in patients with an initial hormonal work-up consistent with a nonfunctioning lesion (9, 21). The main concern in the hormonal follow-up of benign nonfunctioning adrenal tumors is the development of autonomous cortisol secretion without signs of overt Cushing's syndrome (subclinical Cushing’ syndrome) (10, 22). In the present study, dexamethasone suppression and 24h urine collection tests were not performed in the majority of patients for hormonal follow-up. However, a recent report stated that among 63 patients who underwent complete biochemical testing both at baseline and the 2-year follow-up, hormonal hypersecretion was not discovered in any patient at follow-up (20). In addition, the benefit of performing Adx in patients with subclinical Cushing syndrome is controversial (22–26), and conservative treatment could be a good option. At least, for those whose adrenal masses are determined to be nonfunctional at an initial assessment, routine screening including a dexamethasone suppression test and a 24h urine collection test, besides being cumbersome or not being devoid of complications, may provide limited clinical benefits.

There are some limitations of the present study. The evaluation of cases was performed retrospectively, and there was an unavoidable selection bias. The cohort included the patients with short follow-up periods. The cohort included the patients who were referred to urological department for surgery. We excluded patients who were diagnosed as adrenal tumors in the department of endocrinology. Because of those reasons, the percentage of nonfunctioning AIs in all adrenal tumors was not the same as previous reports. The methodologies for radiological and hormonal evaluation were not standardized. Contrast-enhanced CT was not performed in all patients due to renal dysfunction, a history of allergy to contrast agents, or others. Dexamethasone suppression and 24h urine collection tests were not performed for hormonal follow-up in most of our patients, as mentioned above. However, owing to the risk of false-positive results, systemic hormonal follow-up is not recommended in patients with nonfunctioning AIs (5). Lastly, it was difficult to set a clear threshold of the size and an unenhanced CT attenuation value in determining benign AIs in the present study, because they were depending on individual urologist's clinical judgement, personal practice and experience. We included some patients who had small size of AIs less than 1cm in this study, however, cutoff of size for inclusion criteria was unclear for appropriate analysis.

## CONCLUSIONS

We reviewed nonfunctioning AIs in a single center. Repeat radiological examinations demonstrated tumor enlargement in 8.6% of patients who were followed up as nonfunctioning benign AIs. However, tumor enlargement did not correlate with malignancy. The value of repeat radiological and hormonal examinations may be limited in the long-term follow-up of patients whose AIs are not enlarged. To further evaluate the value of long-term follow-up of benign and nonfunctional adrenal tumors, prospective studies are required.

## ABBREVIATIONS

Adx: adrenalectomy
AI: Adrenal incidentaloma
ACTH: adrenocorticotropic hormone
DHEA-S: dehydroepiandrosterone sulphate
CT: computed tomography
HU: hounsfield units
MRI: magnetic resonance imaging
